# Effect of C5a Receptor Antagonist on the Osteogenic Differentiation of Dental Pulp Stem Cells

**DOI:** 10.1002/cre2.70382

**Published:** 2026-05-26

**Authors:** Xiaohan Tan, Junlong Hu, Junhui Li, Zhuangying Chen, Xin Shi, Jie Liu, Mingyue Liu, Weiping Hu

**Affiliations:** ^1^ Department of Prosthodontics the Second Affiliated Hospital of Harbin Medical University Harbin Heilongjiang P.R.China; ^2^ Department of Zhongshan Hospital of Traditional Chinese Medicine Zhongshan Guangdong P.R.China; ^3^ Department of Craniomaxillofacial Surgery Plastic Surgery Hospital, Chinese Academy of Medical Sciences and Peking Union Medical College Beijing P.R.China; ^4^ Department of Prosthodontics the Second Affiliated Hospital of Harbin Medical University& The Key Laboratory of Myocardial Ischemia Ministry of Education Harbin Heilongjiang P.R. China

**Keywords:** C5aR, C5aR antagonist, dental pulp stem cells, osteoblast, osteogenic microenvironment

## Abstract

**Objective:**

Complement component 5a (C5a) binding to its receptor (C5aR) can mediate pro‐inflammatory immune responses, which impairs osteogenic differentiation of dental pulp stem cells (DPSCs). The present study aimed to evaluate the regulatory effect of C5aR antagonist (W54011) on the osteogenic differentiation of DPSCs in an in vitro inflammatory microenvironment and its reparative effect on in vivo LPS‐induced inflammatory mandibular defects, to provide novel strategies for bone regeneration under inflammatory conditions.

**Materials and Methods:**

In vitro, a DPSCs inflammatory model was established with 1.0 μg/mL LPS for 48 h, and cells were divided into four groups (LPS + W54011 + OIM, OIM, W54011 only, blank) with W54011 at 1.0 μg/mL. Each assay included 3 biological and 3 technical replicates, and osteogenic differentiation was assessed via Alkaline Phosphatase (ALP) staining, alizarin red S (ARS) staining, RT‐qPCR, and western blotting for OCN, Osterix, and RUNX2.

In vivo, an LPS‐induced inflammatory mandibular defect model was constructed in Sprague‐Dawley (SD) rats; after 2 weeks of inflammation, defects were implanted with W54011‐loaded Bio‐Oss bone powder, and healing was evaluated by micro‐CT, H‐E staining, and immunohistochemistry for IL‐6, OCN, and RUNX2.

**Results:**

In vitro, all 4 groups showed positive ALP and mineralized nodule staining. Molecular biology methods found differential OCN, Osterix and RUNX2 expression at day 7, 14 and 21, with the LPS + W54011 + OIM group having higher osteogenesis markers at certain times (*p* < 0.05, one‐way ANOVA with Bonferroni post‐hoc test).

In vivo, Micro‐CT and H&E staining revealed that the defect site implanted with W54011‐loaded Bio‐Oss (treatment group) exhibited higher bone density and denser new bone formation compared to the control groups. However, due to the small sample size, these results were considered preliminary qualitative observations without statistical analysis.

**Conclusion:**

1. LPS (1 μg/ml) stimulation for 48 h can be used to establish a DPSCs inflammation model.
2. W54011 can promote the osteogenic differentiation of DPSCs in an inflammatory environment in vitro.
3. Preliminary in vivo data suggest that W54011‐loaded Bio‐Oss may facilitate the healing of inflammatory mandibular defects, warranting further investigation with larger samples.

## 1 Introduction

Complement, an important immune surveillance system, rapidly responds when an immune stimulus occurs and plays an important role in triggering acute inflammation and regeneration of tooth tissue by producing active molecules. It is well known that the complement system can be activated through the classical pathway, the alternative pathway or the lectin pathway and is involved in the body's immune regulation and inflammatory response. After activation of the complement system, complement proteins 3, 4, and 5 and their cleavage products, complement proteins 3a, 4a, and 5a, bind to their corresponding receptors C3aR, C4aR, and C5aR, respectively, to stimulate cell degranulation and release histamine. C3a, C4a, and C5a are collectively referred to as anaphylatoxins, among which C5a has the strongest inflammatory effect, which is 20 times that of C3a and 2500 times that of C4a; therefore, the cascade of complement protein 5 has become the first focus of attention. In recent years, a series of articles by About have been published relating to C5a and DPSCs (Rombouts et al. 2016; Chmilewsky et al. 2013; Giraud et al. 2019). C5a has a role in chemotactic immune cells and DPSCs, and DPSCs can migrate to the injury site along the C5a concentration gradient and differentiate into odontoblasts, finally forming reparative dentin. C5a is also a potent chemokine for neutrophils and other leukocytes and is an important mediator of inflammation (Sumichika et al. 2002). C5a binds to its receptor C5aR on the plasma membrane of its target cells, which can lead to increased intracellular calcium levels and activation of intracellular signaling cascades, which has a cascade effect on immune responses, accompanied by a series of functional responses, such as the recruitment and activation of inflammatory cells, release of granzymes, enhanced expression of cell adhesion molecules, secretion and release of histamine, production of cytokines and chemokines, and vasodilation, which recruits additional immune cells and is not conducive to delaying the immune process, affecting the balance between immunity and regeneration in the tissue. C5a also enhances the immune response, specifically by inducing the secretion of cytokines such as IL1, IL‐6, IL‐8 and TNF‐α (Torgbo 2018) by monocytes, promoting the proliferation of T cells and inducing the production of antibodies by B cells. C5a is one of the most important products of complement activation. After it binds to its corresponding receptor C5aR and is activated, it is involved in the pathological process of many inflammatory diseases, such as cisplatin‐induced glomerulonephritis (Merle et al. 2018), rheumatoid joint inflammation, acute lung injury (Jongerius et al. 2019), sepsis (Qi et al. 2021), atherosclerosis and other cardiovascular system diseases (Niyonzima et al. 2021), as well as the occurrence and development of tumors (Wang et al. 2021a; Afshar‐Kharghan 2017). Therefore, inhibiting the C5a‐C5aR signaling axis can delay the immune process in tissues, which is key to achieving a balance between immunity and regeneration in tissues. In addition, many diseases are caused by complement deficiency. For example, patients with hereditary complement deficiency are susceptible to meningococcal infection. Therefore, our goal is not to control C5a but to inhibit C5a‐C5aR signaling using the W54011. W54011 is an orally active nonpeptide C5a receptor antagonist with high specificity for C5aR that inhibits binding of C5a to the receptor in human neutrophils and hinders the chemotactic response triggered by C5a. W54011 competitively inhibits the effect of C5a at high concentrations but has no effect on C5a expression at low concentrations (Sumichika et al. 2002). Our previous experiments clarified the time and concentration of C5aR antagonists acting on DPSCs. Using C5aR antagonists to block the combination of C5a and C5aR effectively reduces the expression of inflammatory factors, which delays the immune process (this study is being submitted for publication). This not only ensures the regeneration‐promoting effect of C5a but also inhibits the immune process. This study is beneficial for bone regeneration by identifying ways to promote regeneration and suppress inflammation.

Despite the known role of the C5a‐C5aR axis in inflammation and DPSCs biology, the regulatory effect of C5aR antagonists on the osteogenic differentiation of DPSCs in an inflammatory microenvironment remains poorly characterized, and there is a lack of in vivo evidence for the application of W54011 in inflammatory maxillofacial bone defect repair. In addition, most existing studies focus on downregulating C5a levels to block the C5a‐C5aR axis, which may offset the beneficial regenerative effects of C5a on DPSCs. No studies have yet explored the strategy of using C5aR antagonists to selectively block the inflammatory signaling of C5a‐C5aR while preserving C5a's positive regulation of DPSCs regeneration. Based on the above knowledge gap, the central hypothesis of this study is: W54011, a high‐specificity C5aR antagonist, can block the C5a‐C5aR signaling axis to inhibit the local inflammatory response, thereby promoting the osteogenic differentiation of DPSCs in an LPS‐induced inflammatory microenvironment; in addition, W54011‐loaded Bio‐Oss bone powder can improve the healing efficiency of LPS‐induced inflammatory mandibular defects in SD rats by regulating the balance between local inflammation and osteogenesis, without abrogating the regenerative effect of endogenous C5a on DPSCs.

Therefore, this study intended to determine the effect of C5aR antagonists on the osteogenic differentiation of DPSCs and to provide new ideas and approaches for the challenge of osteogenesis occurring in the inflammatory environment.

The regulatory mechanism of DPSCs osteogenic differentiation in the inflammatory microenvironment is closely related to the clinical repair of oral maxillofacial bone defects. DPSCs are easily accessible from extracted wisdom teeth with minimal invasiveness, and they have stronger proliferation and multidirectional differentiation potential than other mesenchymal stem cells (MSCs), making them an ideal seed cell for oral bone tissue engineering. Dental and maxillofacial tissues often suffer from bone defects due to trauma, diseases, and other factors. Since osteogenic differentiation plays a crucial role in repair and regeneration, DPSCs thus become an ideal cell source for research. Clarifying the mechanism of their osteogenic differentiation is helpful for the development of new therapies, such as promoting the repair of oral bone tissues and treating periodontal diseases. This is of great significance for the clinical practice of stomatology and also provides a theoretical basis and cellular support for the development of tissue engineering and regenerative medicine.

## 2 Methods

### 2.1 Summary of DPSCS Culture

Healthy third molars were extracted from 19 ‐to 29 ‐ year‐old patients undergoing mandibular plastic surgery. For each experiment, teeth were obtained from three different donors (*n* = 12; 3–4 third molars per donor; male‐to‐female ratio = 1:1; collection period: September 2020 to December 2021), with each donor serving as an independent biological replicate. All procedures were approved by the Ethics Committee of the Second Affiliated Hospital of Harbin Medical University (ethical approval number: 2022(171)), and written informed consent was obtained from all participants. Extracted teeth were immediately placed in culture flasks containing 5 × antibiotic‐antimycotic solution and stored at 4°C.

On a sterile laminar flow hood, the tooth surface was cleaned of blood stains with alcohol and transferred to a 60 mm Petri dish (Dish 1). Sterile PBS was added to Dish 1 for tooth rinsing. 0.2% Type I collagenase (1 mL per molar) was added to another sterile 35 mm Petri dish (Dish 2). Teeth were longitudinally split using a handpiece to expose the dental pulp (the grinding wheel was avoided from contacting the pulp; teeth were split close to the pulp chamber and then pried open with forceps without touching the pulp).

The dental pulp was separated from the pulp chamber using a sterile dental probe. The apical 1/3 of the pulp was excised with sterile forceps, and the remaining pulp tissue was transferred to Dish. The pulp tissue was minced into 1 mm³ fragments using ophthalmic scissors. The pulp fragments and Type I collagenase in Dish 2 were aspirated into a 15 mL centrifuge tube, which was then placed in a 37°C water bath shaker for 1 h of digestion.

Digestion was terminated by adding 20% fetal bovine serum (FBS) to the centrifuge tube to a final volume of 10 mL. The tube was centrifuged at 1000 rpm for 5 min. After centrifugation, the supernatant was discarded, and 20% FBS was added. Cells were resuspended by pipetting, and this washing‐centrifugation cycle was repeated three times. Following the final centrifugation, the supernatant was removed, and 2 mL of Cyagen cell culture medium was added. The cell suspension was transferred to a culture flask using a pipette.

Culture flasks with seeded cells were incubated in a 37°C incubator with 5% CO₂, and cell adhesion was observed approximately 4 days later. After adhesion, the medium was changed every 2 days. Cell morphology and quantity were monitored using an inverted microscope. When cells reached 80–90% confluence, they were passaged via trypsinization. Only third‐passage DPSCs with stable morphology and proliferation were used for subsequent experiments to ensure experimental consistency.

### 2.2 Treatment Groups and Culture Conditions

Third‐generation pulp cells (all 8 × 10^4^) were cultured in 12 dishes (100 mm) in a 37°C incubator with 5% CO_2_. Culture dishes were randomly assigned to four experimental groups using a random number table to eliminate grouping bias. All cells were washed 3 times with PBS and then incubated in serum‐free DMEM (6 ml/well) for 24 h. The cells were cultured in 1% penicillin, 1% streptomycin, and serum‐free Dulbecco's modified Eagle's medium/F12 (HyClone; GE Healthcare Life Sciences, Logan, UT, USA). The experimental group was designed as follows: (1) LPS + W54011 + OIM: 1.0 μg/ml LPS was added for 48 h, and then 1.0 μg/ml W54011 was cultured in osteogenic induction medium (culture medium contains 10% FBS and supplemented with 0.1 μM dexamethasone, 10 mM β‐glycerol phosphate disodium salt pentahydrate and 50 μg/mL ascorbic acid) for 7, 14 and 21 days. Control groups were designed as follows: (2) OIM: cells were cultured in osteogenic induction medium for 7, 14 and 21 days; (3) W54011 only: 1.0 μg/ml W54011 was added for 7, 14 and 21 days and cells were cultured in osteogenic induction medium (culture medium contains 10% FBS); and (4) Blank: cells were grown in culture media without any other stimulus conditions for 7, 14 and 21 days. Each group included 3 biological replicates (DPSCs from 3 donors) and 3 technical replicates per biological replicate, with the single culture dish as the basic in vitro experimental unit.

### 2.3 Cell Proliferation and Cytotoxicity Assay (CCK‐8 Method)

Cell proliferation and cytotoxicity were evaluated using the Cell Counting Kit‐8 (CCK‐8). According to the negative control requirements of the kit and the grouping design of this study, cells were seeded in a 96 ‐well plate with 2 replicate wells per group, at a density of 5 × 10^3^ cells per well. The 96‐well plate was randomly assigned to seven groups, with the single well as the basic experimental unit. Cells were divided into seven groups as follows:(i) Serum‐free medium containing 1.0 μg/mL W54011 (no cells included, serving as the blank control group);(ii) Medium containing 20% fetal bovine serum (FBS);(iii) Serum‐free medium (control group);(iv) Serum‐free medium containing 0.5 μg/mL W54011;(v) Serum‐free medium containing 1.0 μg/mL W54011;(vi) Serum‐free medium containing 1.5 μg/mL W54011;(vii) Serum‐free medium containing 1.0 μL dimethyl sulfoxide (DMSO).

Cells in all seven groups were cultured in a cell incubator at 37°C with 5% CO _2_ for 24 h and 48 h, respectively. Subsequently, 10 μL of CCK‐8 reagent was added to each well, and the plate was incubated in the 37°C incubator for 2 h and 4 h. After incubation, the plate was placed in a microplate reader, and the absorbance of each well was measured at a wavelength of 450 nm. Results were recorded, colorimetric analysis was performed, and the blank control group was used for zero calibration.

### 2.4 Reverse Transcription‐Quantitative PCR (RT–qPCR)

The expression levels of the genes were quantified using FastStart Universal SYBR‑Green Master mix. Post‐reverse transcription products were used, with two replicate wells set for each group. Each group included 3 biological replicates and 3 technical replicates, with the single PCR reaction well as the technical experimental unit. The reaction system for each well consisted of 3 μL DEPC‐treated water, 5 μL fluorescent dye, 0.5 μL each of forward (F) and reverse (R) primers, and 1 μL cDNA. The RT‐qPCR reaction process was: (1) 95°C for 10 min; (2) 95°C for 30 s; (3) 60°C for 30 s; (4) 72°C for 30 s; (5) return to step 2; (6) for 40 cycles; (7) 72°C for 5 min. RT‐qPCR results were normalized against the reference gene Actin to correct for non‑specific experimental variation. The method of 2^‐ΔΔCt (Livak method) was used to determine the relative quantity of mRNA expression in the sample. The primers were used as described in the RT‐PCR method. The following primers were used: OCN forward,5′‐CACTCCTCGCCCTATTGGC‐3′and reverse, 5′‐CCCTCCTGCTTGGACACAAAG‐3′; Osterix forward, 5′‐CCTCTGCGGGACTCAACAAC‐3′ and reverse, 5′‐AGCCCATTAGTGCTTGTAAAGG‐3′; RUNX2 forward, 5′‐CCGCCTCAGTGATTTAGGGC‐3′ and reverse, 5′‐GGGTCTGTAATCTGACTCTGTCC‐3′.

Relative mRNA expression data were tested for normality (Shapiro‐Wilk test) and analyzed by one‐way ANOVA with Bonferroni post‐hoc test (*p* < 0.05 for statistical significance); data are presented as mean ± standard deviation (SD).

### 2.5 Western Blotting

Cells (6 × 10^6^ cells/dish) were grown to confluence in a 100 mm dish and lysed in RIPA lysis buffer containing 1% PMSF for 30 min. After the cell lysates were clarified by centrifugation at 13,300 × g for 15 min at 4°C to remove cell debris, the total protein content was measured using a Pierce™ BCA Protein Assay kit according to the manufacturer's protocol. We added 10 μg of protein lysate to each lane of the gel separately. Ten percent SDS–PAGE was prepared for RUNX2, Osterix and actin antibodies, and 15% SDS–PAGE was prepared for OCN antibody. Subsequently, the membranes were blocked in 5% skim milk for 1 h with gentle agitation at room temperature. The membranes were then incubated at 4°C overnight with the primary antibodies: OCN polyclonal antibody rabbit polyclonal (cat. ab133612; 1:1,000; Abcam, Britain), Osterix (cat. ab209484; 1:1,000; Abcam, Britain), RUNX2 (cat. ab192256; 1:1,000; Abcam, Britain) and β‐actin (cat. No. 20536‐1‐ AP; 1:5,000; ProteinTech, China). Following primary antibody incubation, the membranes were incubated with the following secondary antibodies at room temperature for 1 h: goat anti‐mouse IgG (cat. No. SA00001‐1; 1:5000; ProteinTech Group Inc.) and goat anti‐rabbit IgG (cat. No. SA00001‐2; 1:10,000; ProteinTech Group Inc.). Blue Plus® IV Protein Marker (10‐180 kDa, cat. No. DM131‐01, TransGen Biotech) was used to monitor the electrophoresis and membrane transfer. Bands were visualized using the Ultrasensitive ECL Detection Kit (PK10003, ProteinTech Group Inc.) and photographed using the Tanon 1000 digital image gel analytical system (Tanon Science & Technology Co. Ltd.). β‐actin was used as a loading control. Bands were quantified using ImageJ (NIH, USA). Three biological replicates were set up for each group, with each biological replicate containing three technical replicates; the single protein sample lane was the technical experimental unit. Protein gray value data were normalized to β‐actin, tested for normality (Shapiro‐Wilk test), and analyzed by one‐way ANOVA with Bonferroni post‐hoc test (*p* < 0.05 for statistical significance).

### 2.6 Alkaline Phosphatase Stain

After cells were induced and cultured for 7, 14 and 21 days, DPSCs (2 × 10 ^5^ cells/well) were fixed in 4% paraformaldehyde for 30 min. Detection was performed by the BCIP/NBT Alkaline Phosphatase Color Development Kit (Beyotime Institute of Biotechnology, China). According to the manufacturer's instructions, each group of cells was stained for 6 min. Bands were then recorded by a digital camera. Three biological replicates were set up for each group, with each biological replicate containing three technical replicates. ALP staining images were semi‐quantitatively analyzed for positive staining area using ImageJ software; data were processed with normality test and one‐way ANOVA with Bonferroni post‐hoc test (*p* < 0.05 for statistical significance).

### 2.7 Mineralization Induction

Cells (2 × 10 ^5^ cells/well) were seeded and cultured for 7, 14 and 21 days. After the cells were induced and differentiated, the culture medium was removed. Then, the cells were washed with PBS once. According to the manufacturer's instructions, the cells were fixed for 20 min and stained with Alizarin red S staining solution (C0148S, Beyotime, China) for 30 min. Bands were then recorded using a digital camera. Three biological replicates were set up for each group, with each biological replicate containing three technical replicates. Alizarin red S staining was semi‐quantitatively analyzed for mineralized nodule number and positive area using ImageJ software; data were tested for normality and analyzed by one‐way ANOVA with Bonferroni post‐hoc test (*p* < 0.05 for statistical significance).

### 2.8 Preparation and Grouping of Mandibular Defect Model in SD Rats

The animal experiments in this study were approved by the Animal Ethics Committee of the Second Affiliated Hospital of Harbin Medical University (approval number ky2020‐057), and all experimental procedures were performed in strict accordance with the Guidelines for Ethical Review of Laboratory Animal Welfare (Torgbo, 35892‐2018) and the 3 R principle (Replacement, Reduction, Refinement) of laboratory animal care. Sample size (n = 3 per group) was determined based on pre‐experiment results, which confirmed that this sample size could detect significant phenotypic differences in bone repair and inflammation levels, while minimizing the number of experimental animals used. SD rats were injected intraperitoneally with 0.3 mL/100 g of 10% chloral hydrate (chloral hydrate dose/rat body weight), and the limbs of the rats were fixed on the operating table. The subcutaneous tissue was incised along the lower edge of the mandible of the rat, and the deep fascia and periosteum between the occlusal and diastasis muscles were bluntly separated to fully expose the bone surface of the mandibular region of the anterior teeth. A parallel line 0.5 mm above the mandibular inferior border was defined as the base line of the mandibular defect, and a 6 × 2 mm mandibular defect model was prepared using a 1.2 mm diameter flat‐ended cylindrical diamond turning needle.

Twelve healthy male SD rats of 10 weeks of age, weighing about 300 g, were taken and randomly assigned into the following four groups (n = 3 per group) using a random number table, with each rat serving as an independent in vivo experimental unit: A. Sham group: cut the skin, bluntly separate the periosteum, storm the bone tissue, reposition the subcutaneous tissue and then tightly suture, 2 weeks later to take the material; B. Inflammation group: construct a 6 × 2 × 0.5 mm mandibular defect model, apply 5 μl of LPS solution at a concentration of 4 μg/ml, locate the subcutaneous tissue and then close it tightly with sutures, and inject 5 μl of 4 μg/ml LPS solution every other day, and then take the material after 2 weeks; C. Inflammation + bone powder group: construct a 6 × 2 × 0.5 mm mandibular defect model, apply 5 μl of LPS solution at a concentration of 4 μg/ml, locate the subcutaneous tissue and then suture it tightly, inject 5 μl of 4 μg/ml LPS solution every other day, and then expose the mandibular defect 2 weeks later, place the Bio‐Oss bone powder soaked in sterile saline, suture the subcutaneous tissue, and then take the material 2 weeks later; D. Treatment group: construct a 6 × 2 × 0.5 mm mandibular defect model, apply 5 μl of LPS solution at a concentration of 4 μg/ml, locate the subcutaneous tissues and then tightly suture them, inject 5 μl of 4 μg/ml LPS solution every other day, expose the mandibular defect 2 weeks later, place Bio‐Oss bone powder impregnated with 1 μg/ml W54011 solution, sew up the subcutaneous tissues, and take the material 2 weeks later.

### 2.9 Micro‐CT Analysis of Mandibular Defect Volume

To determine the available mandible bone volume, 12 SD rats were euthanized by intraperitoneal injection of sodium pentobarbital (150 mg/kg), and their mandibles were collected for further micro‐CT analysis, with projection images of the mandibles obtained through a micro‐CT scanner (SCANCOlCT50, Switzerland); alternatively, rats were euthanized by intraperitoneal injection of sodium pentobarbital (150 mg/kg) to collect mandible samples, which were first fixed in 20 ml of 4% paraformaldehyde for 24 h, then mandible specimen images were acquired by Micro‐CT (Micro‐Computed Tomography) with device resolution of 10 μm, source voltage of 90 kV and current of 200 µA, and mandibular specimen reconstruction was performed via 2D and 3D images.

### 2.10 H‐E Dye Staining Procedure for Rat Mandible Tissue Sections

The rat mandible was collected, washed with saline, and fixed in 4% paraformaldehyde at 4°C for 24 h. After PBS washing, the sample was decalcified in 10% EDTA (pH 7.2) at room temperature, with the solution replaced every 2 days until complete decalcification (confirmed by needle penetration). The bone tissue was then trimmed, rinsed with distilled water, dehydrated in an ethanol series, and cleared in xylene. Subsequently, the tissue was embedded in molten paraffin, cooled, and sectioned into 4‐5 µm slices, which were mounted on adhesive slides and dried. After dewaxing in xylene and rehydration in ethanol, hematoxylin‐eosin (H&E) staining was performed. Finally, the sections were dehydrated, cleared in xylene, mounted with neutral gum, and observed under a microscope to assess osteogenesis, with images captured for analysis.

### 2.11 Immunohistochemical Staining Procedure for Rat Paraffin Sections

The paraffin‐embedded rat sections were baked at 58°C for 2 h for dewaxing, followed by xylene treatment (three times, 10 min each) and hydration in a graded ethanol series (100%, 95%, and 80%, 5 min each). After PBS washing (3 × 5 min), slides were treated with cold acetone at −20°C for 15 min, washed again, and endogenous peroxidase was blocked with 0.3% H_2_O_2_ in methanol (4°C, 15 min). Following PBS washes, membrane permeabilization was performed with 0.1% Triton X‐100 (4°C, 15 min). After blocking with normal goat serum (RT, 20 min), primary antibody (50 µL) was applied overnight at 4°C or for 1 h at RT. Slides were washed, incubated with secondary antibody (40‐50 µL, 1 h at RT), and developed with DAB (5‐10 min, monitored microscopically). Color development was stopped with deionized water, followed by hematoxylin counterstaining (2‐5 min), differentiation in HCl‐alcohol, and bluing in tap water. Finally, sections were dehydrated (80%, 95%, and 100% ethanol), cleared in xylene (2 × 3 min), mounted, and examined under a microscope. Positive staining of IL‐6, OCN and RUNX2 was semi‐quantitatively analyzed for positive area and optical density using ImageJ software; data are presented as preliminary observations due to the small sample size (n = 3) without statistical analysis.

### 2.12 Identification of DPSCs By Flow Cytometry with CD34, CD45, CD73, CD90, and CD105

DPSCs cultured in seven 75 cm² flasks were prepared for identification: washed 3 times with PBS, digested with 2 ml pre‐warmed 0.25% trypsin for collection, then counted after terminating digestion. Cells at 1×10⁶ cells/ml were centrifuged in 15 ml tubes, rinsed with PBS (300 g, 10 min), supernatant aspirated completely, and the pellet resuspended in 150 μl 3% BSA for Fc receptor blocking at room temperature for 10 min. Specific antibodies against human surface antigens (APC anti‐human CD45, FITC anti‐human CD90 (Thy1), PE/Cyanine7 anti‐human CD73 (Ecto‐5’‐nucleotidase), PE anti‐human CD105 (Endoglin), PerCP/Cyanine5.5 anti‐human CD34) and PE/FITC‐conjugated IgG isotype controls (1:10 dilution, 5 μl antibody + 50 μl buffer per 1 × 10⁶ cells) were prepared. Appropriate antibodies were added to tubes: one as isotype control, others incubated with monoclonal conjugated antibodies at 2°C‐8°C for 10 min in the dark (note: prolonged high‐temperature incubation causes non‐specific labeling; extend time if on ice). Cells were washed with 1‐2 ml PBS, centrifuged (300 rpm, 10 min), supernatant removed, and resuspended in washing solution for flow cytometry analysis.

Flow cytometry data were analyzed using FlowJo software, with the positive expression rate of surface markers calculated as the percentage of positive cells in the total cell population; three biological replicates were performed, and results are presented as mean ± SD.

## 3 Results

### 3.1 Cell Morphology and Identification of DPSCs

**Figure 1:**
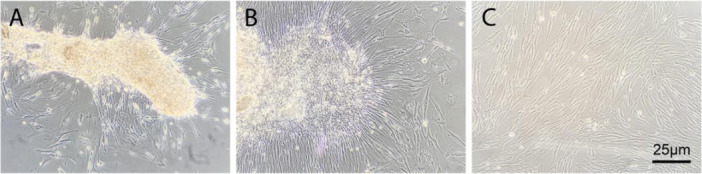
Morphological characteristics of DPSCs. (A) Adherent DPSCs at 4 days of primary culture; (B) Fibroblast‐like DPSCs with uniform morphology at 2 weeks of culture; (C) Actively proliferating DPSCs after passage (magnification, × 10; scale bar, 25 μm).

The cell morphology of primary DPSCs was assessed and is shown in Figure 1. Primary culture DPSCs adhered to the wall of the culture flask on day 4 (Figure 1A) and then generated clonogenic cell populations by day 7. Cells were spindle‐shaped with a typical fibroblast‐like morphology. After 14 days of culture, the cell morphology was more uniform, with fibroblast morphology and long cell processes arranged in parallel and reaching a sufficient number for passage (Figure 1B). After passage, the cells proliferated rapidly (Figure 1C).

### 3.2 Cell Proliferation and Cytotoxicity Assay (CCK‐8 Method)

**Figure 2:**
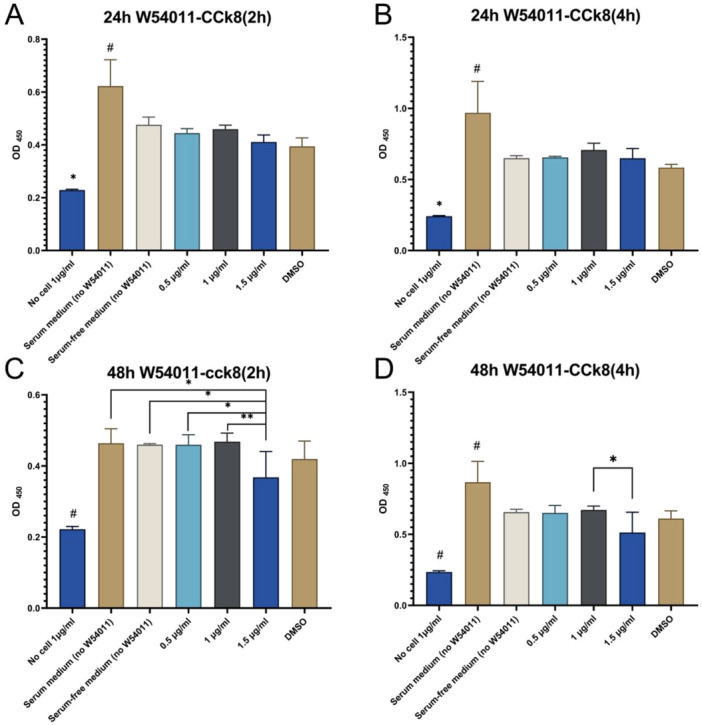
CCK8 cell proliferation assay. 24 and 48 h after W54011 treated on dental pulp cells. (A) (B) #*p* < 0.05 versus other groups; *p < 0.05 versus other groups. (C) #*p* < 0.05 versus other groups; *p < 0.05, **p < 0.01 versus 1.5 μg/ml W54011 groups. (D) #*p* < 0.05 versus other groups; *p < 0.05 versus 1.5 μg/ml W54011 groups. Data are presented as mean ± SD.

Dental pulp cells were cultured to the third passage and treated with 0.5 μg/ml, 1.0 μg/ml, and 1.5 μg/ml of C5aR antagonist (W54011) for 24 h and 48 h, respectively, followed by the detection of cell proliferation. The results showed that at 24 h after W54011 treatment, the cell proliferation efficiency of the three W54011 concentration groups under 2 ‐hour and 4 ‐hour CCK‐ 8 incubation was basically consistent with that of the control group (serum‐free medium + cells) (*p* > 0.05). However, under the conditions of 2‐h and 4‐h CCK‐8 incubation combined with 48‐h W54011 treatment, the 1.5 μg/ml W54011 group exhibited a significant inhibitory effect on cell proliferation compared with other groups (*p* < 0.05). The cell growth of the 1.0 μg/ml W54011 group remained similar to that of the control group throughout the experiment (*p* > 0.05) (Figure 2). No significant cytotoxicity was observed in the 0.5 μg/mL and 1.0 μg/mL W54011 groups, confirming 1.0 μg/mL as the optimal safe concentration for subsequent experiments.

### 3.3 Cell Morphology and Growth in Different Groups

**Figure 3:**
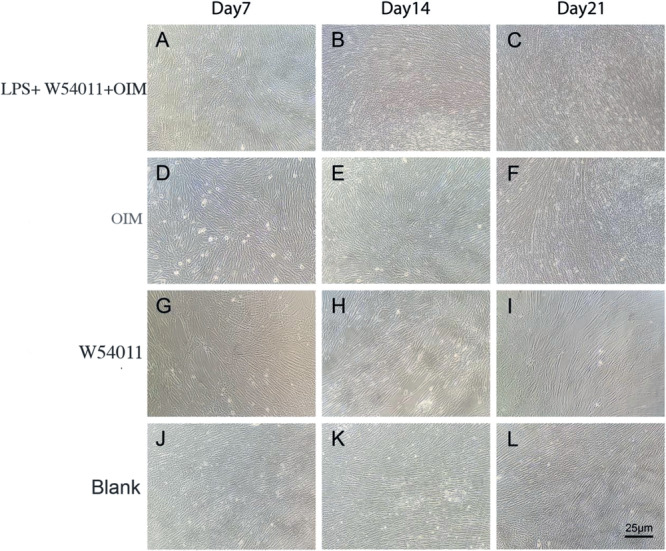
Morphological appearance of DPSCs cultured with W54011 (magnification, × 10; scale bar, 25 μm). DPSCs were cultured in osteogenic induction medium and normal medium with 10% serum for 7, 14, and 21 days. (A) Experimental group, 1.0 μg/ml W54011 in osteogenic induction medium for 7 days. (B) Experimental group, 1.0 μg/ml W54011 in osteogenic induction medium for 14 days. (C) Experimental group, 1.0 μg/ml W54011 in osteogenic induction medium for 21 days. (D) Control group, osteogenic‐induced medium for 7 days. (E) Control group, osteogenic‐induced medium for 14 days. (F) Control group, osteogenic‐induced medium for 21 days. (G) Treatment group, 1.0 μg/ml W54011 for 7 days. (H) Treatment group, 1.0 μg/ml W54011 for 14 days. (I) Treatment group, 1.0 μg/ml W54011 for 21 days. (J) Blank control, normal medium with 10% serum for 7 days. (K) Blank control, normal medium with 10% serum for 14 days; (L) Blank control, normal medium with 10% serum for 21 days.

The cell morphology of cultured DPSCs in different groups was assessed (Figure 3). After DPSCs were cultured for 7 days, the morphology of cells in each group was normal in shape and closely arranged, and there was no significant difference among the groups (Figure 3A,D,G,J) (*p* > 0.05). After 14 days of culture, cells in the LPS + W54011 + OIM and control groups were tightly packed, some cells were polygonal and pyramidal in shape, and osteoblast‐like changes appeared (Figure 3B,E). The cells in the W54011 only group decreased in number and density and maintained normal morphology (Figure 3H). The cell shape in the blank control group was primarily long spindles and close‐packed‐like fences (Figure 3K). After 21 days in culture, there were increased numbers of polygonal and pyramidal cells in the LPS + W54011 + OIM and control groups, and the multilayered cells formed nodular crystals (Figure 3C,F). The number and density in only the W54011 group decreased, and the cell morphology changed abnormally (Figure 3I). Cells in the blank control group were primarily fusiform and arranged in a palisade (Figure 3L). Osteoblast‐like morphological changes in the LPS + W54011 + OIM group were consistent with the results of osteogenic marker detection, suggesting that W54011 promotes osteogenic morphological transformation of DPSCs in an inflammatory microenvironment.

Osteogenic induction medium and normal medium with 10% serum scaffolds for 7, 14 and 21 days. (A) Experimental group, 1.0 μg/ml W54011 in osteogenic inductionmedium for 7 days; (B) Experimental group, 1.0 μg/ml W54011 in osteogenic induction medium for 14 days; (C) Experimental group, 1.0 μg/ml W54011 in osteogenic induction medium for 21 days; (D) Control group, osteogenic‐induced medium for 7 days; (E) Control group, osteogenic‐induced medium for 14 days; (F) Control group, osteogenic‐induced medium for 21 days; (G) Treatment group, 1.0 μg/ml W54011 for 7 days; (H) Treatment group, 1.0 μg/ml W54011 for 14 days; (I) Treatment group, 1.0 μg/ml W54011 for 21 days; (J) Blank control, normal medium with 10% serum for 7 days; (K) Blank control, normal medium with 10% serum for 14 days; (L) Blank control, normal medium with 10% serum for 21 days.

### 3.4 Reverse Transcription‐Quantitative PCR (RT–qPCR)

**Figure 4:**
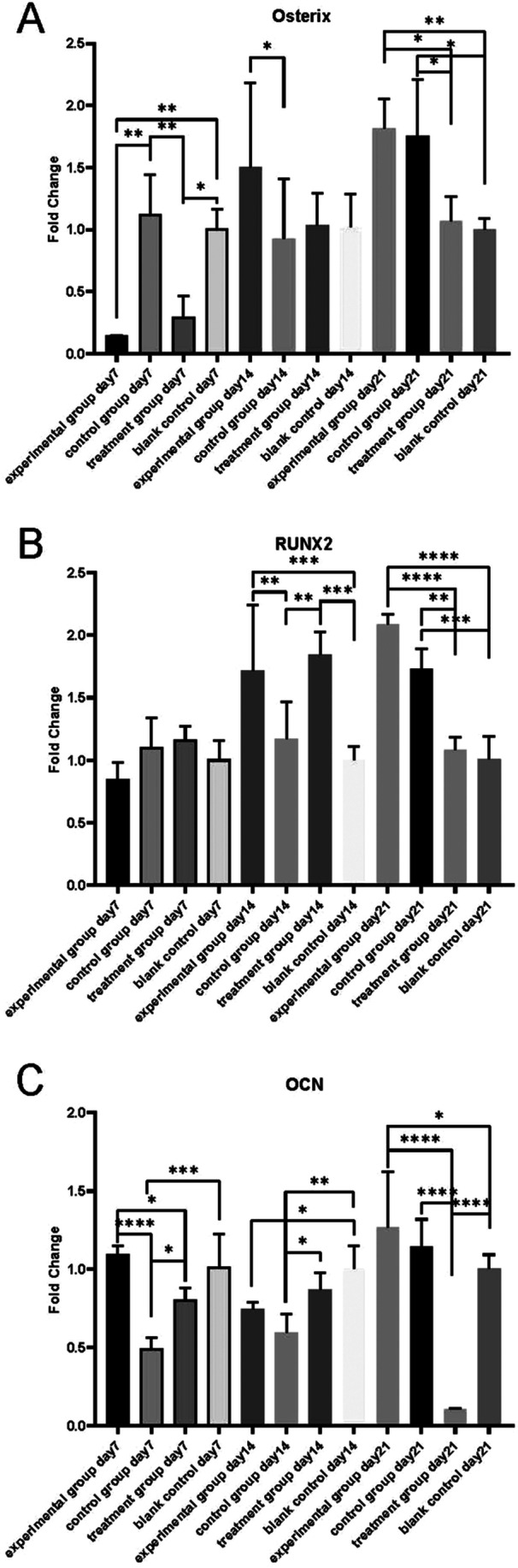
Relative mRNA expression levels of Osterix, RUNX2, OCN detected by RT‐qPCR. (A) Osterix (B) RUNX2 (C) OCN. Averaged the Ct values and calculated the values per sample as following formula: ΔCt treated = Ct reference gene, treated – Ct target gene, treated. ΔCt control = Ct reference gene, control – Ct target gene, control. Then use standard methods to estimate the mean difference, this mean difference between these ΔCt values is ΔΔCt = mean (ΔCt treated) – mean (ΔCt control). And the fold change was determined as 2‐ΔΔCt. * means *p* < 0.05 compared with other groups; ** means *p* < 0.01; *** means *p* < 0.001; **** means *p* < 0.0001. Data are presented as mean ± SD; statistical analysis: Shapiro‐Wilk normality test + one‐way ANOVA with Bonferroni post‐hoc test.

Osterix gene expression was lowest in the experimental group, followed by the W54011 only group on day 7 (*p* < 0.05). On day 14, the experimental group expressed higher levels than the control group (*p* < 0.05). The experimental group and the W54011 only group expressed the RUNX2 gene at higher levels than the control and blank control groups on day 14 (*p* < 0.05) (Figure 4C). There was no significant difference in either Osterix or RUNX2 gene expression between the experimental group and the control group or between the W54011 group and the blank control group on day 21 (*p* > 0.05) (Figure 4A,B). However, both the experimental group and the control group had higher Osterix and RUNX2 expression than the W54011‐only group and the blank control group on day 21 (*p* < 0.05).

The experimental group and the blank control group expressed higher levels of the OCN gene than the other 2 groups on day 7 (*p* < 0.05), and the control group expressed the lowest levels of all 4 groups (*p* < 0.05). The blank control group expressed the highest OCN on day 14 compared with the other 3 groups (*p* < 0.05). OCN gene expression was highest in the experimental group and lowest in the W54011 only group on day 21 (*p* < 0.05). These results indicated that W54011 could upregulate the transcription of core osteogenic genes in DPSCs at the late stage of inflammatory induction (day 14 and 21), which is the key period of osteogenic differentiation.

### 3.5 Western Blotting

**Figure 5:**
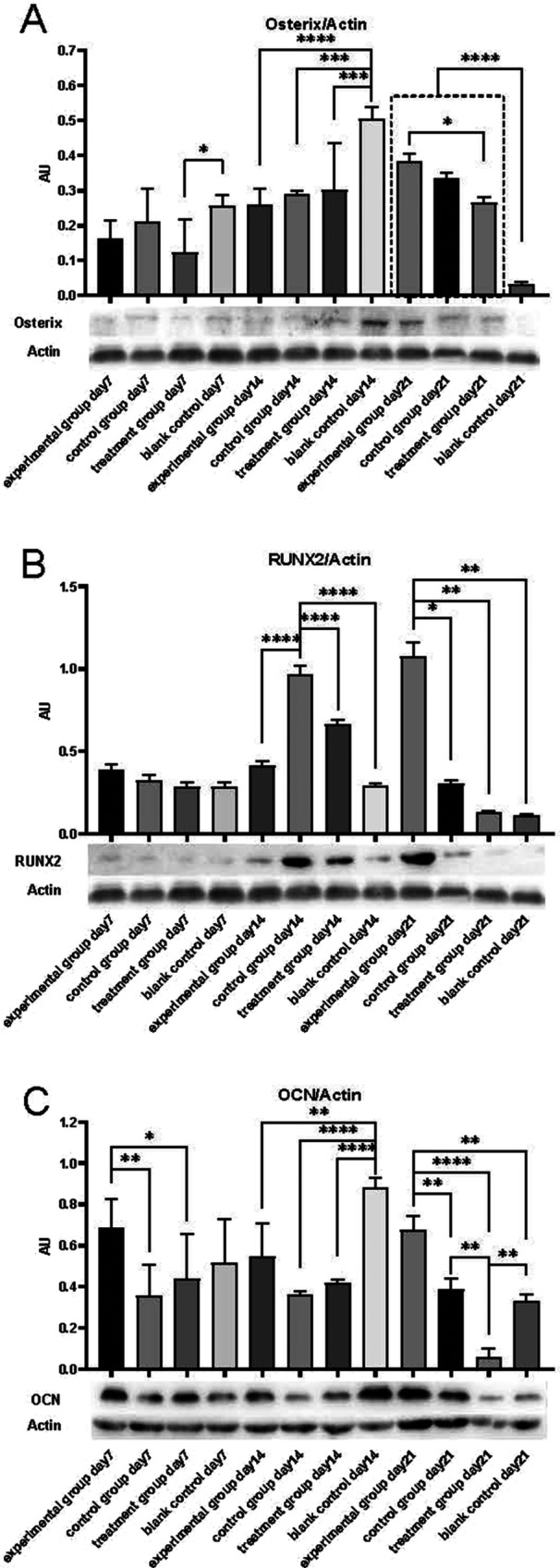
OCN, Osterix, RUNX2 and Actin expression detected by western blotting analysis (*n* = 3). (A) Osterix (B) RUNX2 (C) OCN.

Western blotting analysis revealed that the LPS + W54011 + OIM group expressed higher OCN protein levels than the OIM group and the W54011 only group, but there was no difference between the LPS + W54011 + OIM and blank control groups on day 7 (*p* < 0.05). The blank control group expressed the highest OCN protein level compared with the other groups on day 14 (*p* < 0.05). The LPS + W54011 + OIM group expressed the highest level of OCN protein, and the W54011 group expressed the lowest level of OCN protein on day 21 (*p* < 0.05) (Figure 5C). There was no difference in Osterix and RUNX2 expression on day 7 (*p* > 0.05). The blank control expressed higher levels of Osterixes than the other groups on day 14 but expressed the lowest levels on day 21. The OIM group expressed higher RUNX2 than the other groups on day 14 (*p* < 0.05) and lower RUNX2 than the LPS + W54011 + OIM group on day 21 (*p* < 0.05) (Figure 5A,B). Protein expression results were consistent with RT‐qPCR data, confirming that W54011 promotes the translation of osteogenic markers in DPSCs at the late stage of inflammatory osteogenic induction, with the most significant effect on OCN (a late osteogenic marker).

Determine the background‐subtracted densities of the protein of interest (PI) and the normalizing control (NC). Identify the NC that has the highest density value. Divide all the NC values by the highest NC density value to get a relative NC value. Divide all the PI values by the relative NC values in their respective lanes. ‐‐means *p* < 0.05 compared with other groups; ** means *p* < 0.01; *** means *p* < 0.001; **** means *p* < 0.0001. Data are presented as mean ± SD; statistical analysis: Shapiro‐Wilk normality test + one‐way ANOVA with Bonferroni post‐hoc test.

### 3.6 Alkaline Phosphatase Staining

**Figure 6:**
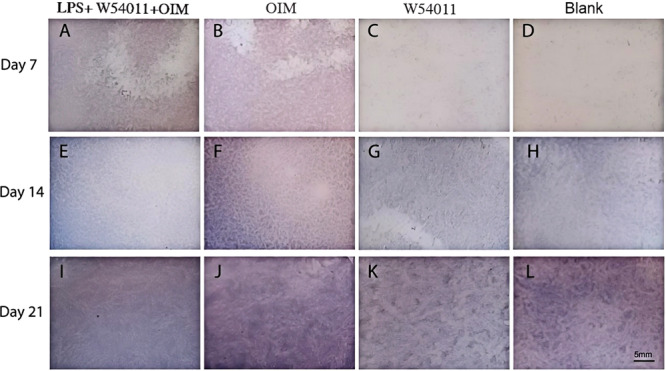
ALP activity quantitation of HDPSCs grown on W54011, osteogenic induction medium and normal medium with 10% serum for 7, 14 and 21 days (magnification, × 10; scale bar, 5 mm).

To determine the mineralization capacity of DPSCs in different groups, ALP staining was performed on cells cultured for 7,14 and 21 days (*p* < 0.05) (Figure 6). ALP staining in each group is shown in blue. ALP activity increased with increasing culture time in each group. When compared to the LPS + W54011 + OIM group with the same incubation time, the OIM groups exhibited higher ALP activity, and the W54011 only and blank control groups were reduced. Furthermore, the highest activity was observed on OIM group day 21, and the lowest activity was observed in the blank control on day 14 (*p* < 0.05) (Figure 6H).

ALP activity of the cells grown on LPS + W54011 + OIM group and control group was significantly higher than that on treatment group and blank control at day 7,14 and 21. (A) LPS + W54011 + OIM group, 1.0 μg/ml W54011 in osteogenic induction medium for 7 days; (B) Control group, osteogenic‐induced medium for 7 days; (C) Treatment group, 1.0 μg/ml W54011 for 7 days; (D) Blank control, normal medium with 10% serum for 7 days; (E) Experimental group, 1.0 μg/ml in osteogenic induction medium for 14 days; (F) Control group, osteogenic‐induced medium for 14 days; (G) Treatment group, 1.0 μg/ml for 14 days; (H) Blank control, normal medium with 10% serum for 14 days; (I) Experimental group, 1.0 μg/ml W54011 in osteogenic induction medium for 21 days; (J) Control group, osteogenic‐induced medium for 21 days; (K) Treatment group, 1.0 μg/ml W54011 for 21 days; (L) Blank control, normal medium with 10% serum for 21 days.

### 3.7 Mineralized Nodule Staining

**Figure 7:**
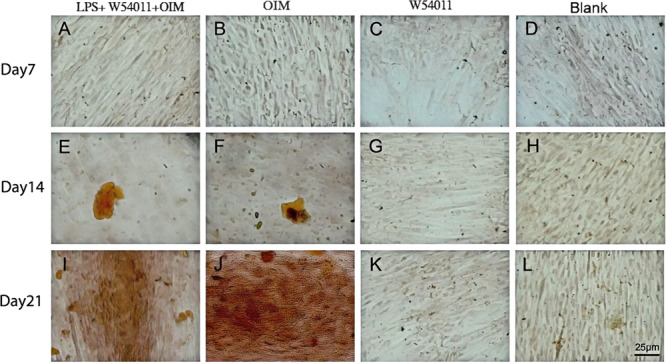
Staining results of mineralized nodules were detected in different groups (magnification, × 40; scale bar, 25 μm).

The cells adopted an osteoblast‐like polygonal morphology after 21 days of culture. Mineralized nodules were further evaluated using Alizarin red S (ARS) staining to monitor the degree of mineralization (Figure 7). Mineralized nodules became red mineralized nodules following staining with Alizarin Red S. Much stronger Alizarin Red staining was seen when the DPSCs were grown with osteogenic induction medium than that seen in the cells grown with ordinary culture medium.

(A) LPS + W54011 + OIM group, 1.0 μg/ml W54011 in osteogenic induction medium for 7 days; (B) Control group, osteogenic‐induced medium for 7 days; (C) Treatment group, 1.0 μg/ml W54011 for 7 days; (D) Blank control, normal medium with 10% serum for 7 days; (E) LPS + W54011 + OIM group, 1.0 μg/ml W54011 in osteogenic induction medium for 14 days; (F) Control group, osteogenic‐induced medium for 14 days; (G) Treatment group, 1.0 μg/ml W54011 for 14 days; (H) Blank control, normal medium with 10% serum for 14 days; (I) LPS + W54011 group, 1.0 μg/ml W54011 in osteogenic induction medium for 21 days; (J) Control group, osteogenic‐induced medium for 21 days; (K) Treatment group, 1.0 μg/ml W54011 for 21 days; (L) Blank control, normal medium with 10% serum for 21 days. Data are presented as mean ± SD; n = 3 biological replicates, 3 technical replicates per group; statistical analysis: Shapiro–Wilk normality test + one‐way ANOVA with Bonferroni post‐hoc test.

### 3.8 Large‐Scale Observations of the Rats

**Figure 8:**
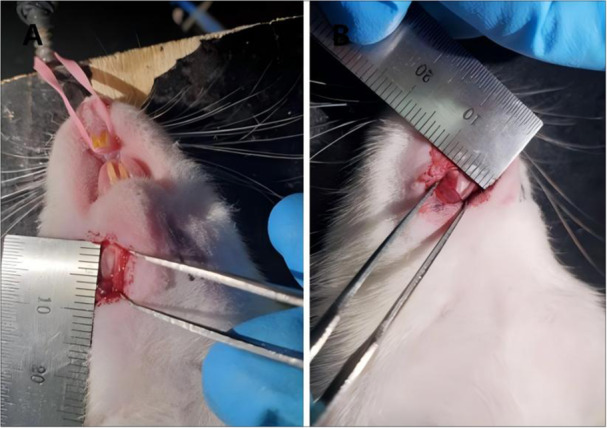
Preparation of mandibular defects in SD rats during surgery. (A) The mandibular defect of SD rats is 6 mm long; (B) the mandibular defect of SD rats is 2 mm wide.

A total of 12 SD rats were divided into 4 groups and all survived without accidental death. Among them, the rats in the inflammation group and the inflammation + bone powder group had decreased body weight and decreased activity. The 6 × 2 × 0.5 mm, mandibular defect model can be successfully prepared (Figure 8).

### 3.9 3D Reconstruction and Analysis of Micro‐CT

**Figure 9:**
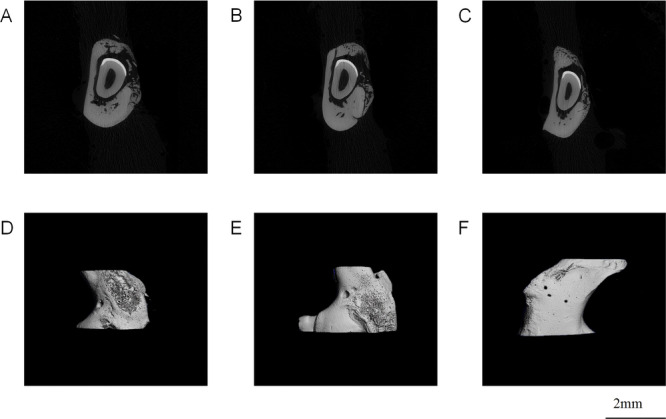
Micro‐CT observed the healing of the mandibular defects in SD rats(scale bar, 2 mm). (A, D) inflammation group; (B, E) Inflammation + bone powder group; (C, F) treatment group.

The healing of mandibular defects in SD rats was observed using Micro‐CT. In the inflammatory group, mandible defects were obvious with poor healing and low trabecular density (Figure 9A,D). The inflammation + bone powder group healed better, and discontinuous new bone tissue was visible in the defect (Figure 9B,E). The mandible defect in the treated group healed well with high bone density, and discontinuous cortical bone was visible in cross section (Figure 9C,F).

### 3.10 H–E Histological Observation

**Figure 10:**
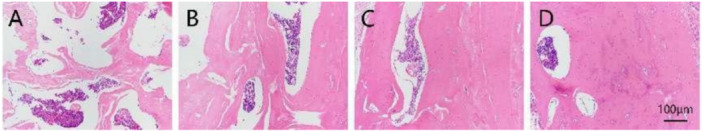
Bone healing status observed by H‐E histology (magnification, × 40; scale bar, 100μm). (A) Inflammation group; (B) Inflammation + bone powder group; (C) Treatment group; (D) Normal mandibular bone tissue. Results are described as preliminary observations due to small sample size (n = 3).

H‐E histological observation revealed a small number of disordered trabecular bone and extensive inflammatory cell infiltration in the inflammation group (Figure 10A); the inflammation + bone powder group showed more orderly trabecular bone, increased new bone tissue and reduced inflammatory cell infiltration (Figure 10B); the treatment group formed denser bone tissue with a regular fibrous structure, abundant cell nuclei, active bone reconstruction, continuous dense bone matrix and mature osteoblasts (Figure 10C); the normal rat mandible showed intact cortical bone and regular trabecular bone with no inflammatory cell infiltration (Figure 10D).

### 3.11 IL‐6, OCN, and RUNX2 Proteins Were Detected By Immunohistochemistry

**Figure 11:**
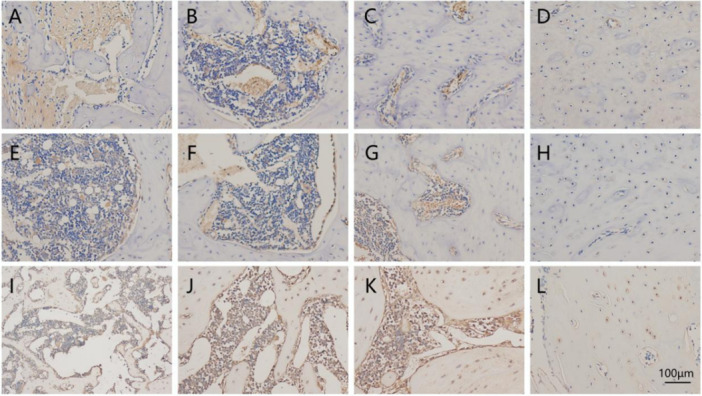
Immunohistochemical detection of IL‐6, OCN, and RUNX2 protein expression (magnification, × 40; scale bar, 100μm). (A) IL‐6 expression in the inflammation group; (B) IL‐6 expression in the inflammation + bone powder group; (C) IL‐6 expression in the treatment group; (D) IL‐6 expression in the sham ‐operated group; (E) OCN expression in the inflammation group; (F) OCN expression in the inflammation + bone powder group; (G) OCN expression in the treatment group; (H) OCN expression in the sham‐operated group; (I) RUNX2 expression in the inflammation group; (J) RUNX2 expression in the inflammation + bone powder group; (K) RUNX2 expression in the treatment group; (L) RUNX2 expression in the sham‐operated group. Results are described as preliminary observations due to small sample size (n = 3).

IL‐6 expression: the positive expression was highest in the inflammation group, the trace expression in the sham group, and the treatment group was lower than that in the inflammation + bone powder group (Figure 11A–D).

OCN expression: OCN positive cells compared with other groups, and the expression of inflammation + bone powder group was higher than the inflammation group and the sham group (Figure 11E–H).

RUNX2 Expression: RUNX2 positive cells compared with other groups, while the inflammatory group showed the least expression (Figure 11I–L).

### 3.12 Identification of Dpscs By CD34, CD45, CD73, CD90 and CD105 Using Flow Cytometry

**Figure 12:**
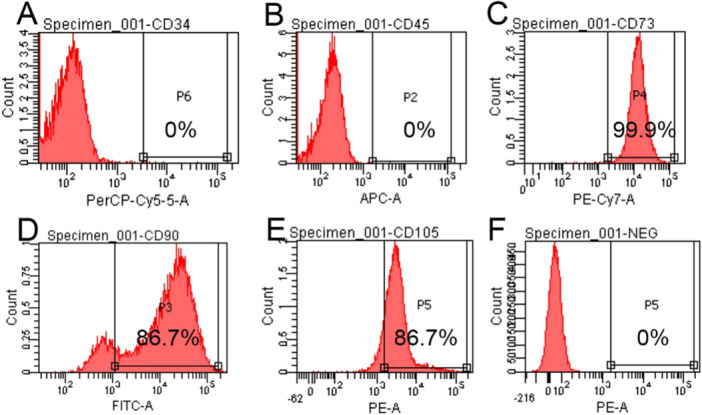
Expression profiles of stem cell surface markers in human DPSCs cultured with Mesenchymal Stem Cell Growth Medium, as determined by FAC flow cytometry. (A) CD34; (B) CD45; (C) CD73; (D) CD90; (E) CD105; (F) Isotype control (NEG); The positive expression rate is the percentage of positive cells in the total cell population (mean ± SD, n = 3).

Flow cytometry analysis showed that the expression levels of CD34 (Figure 12A), CD45 (Figure 12B), CD73 (Figure 12C), CD90 (Figure 12D), CD105 (Figure 12E), and isotype control (NEG, Figure 12F) in DPSCs were 0.1%, 0%, 99.9%, 86.7%, 86.7%, and 0%, respectively. DPSCs positively expressed the mesenchymal stem cell surface markers CD73, CD90, and CD105, while the hematopoietic stem cell markers CD45 and CD34 were negatively expressed, consistent with the phenotypic characteristics of mesenchymal stem cells.

## 4 Discussion

### 4.1 Summary of Core Research Findings

After plastic revision surgery, poor bone formation caused by inflammation impairs surgical outcomes (Schmidt‐Bleek et al. 2015; Seebach and Kubatzky 2019), and only inflammation‐resistant bone formation is considered successful. This study systematically explored the effects of the W54011 on osteogenic differentiation of DPSCs and mandibular bone defect repair through in vitro and in vivo experiments, with the following core results.

In vitro experiments: An inflammatory DPSCs model was established using LPS. The results showed that W54011 had no inhibitory effect on osteogenic differentiation; the LPS + W54011 + OIM group maintained osteogenesis‐related factor expression similar to the OIM‐only group. Notably, the W54011‐only group also exhibited comparable expression of Osterix, OCN, and RUNX2 compared to the normal mineralized medium control group. ALP staining and Alizarin Red further confirmed that W54011 did not affect DPSCs differentiation into osteoblasts. Additionally, this study dynamically evaluated osteogenic gene expression at three key time points, which is more rigorous than single‐time‐point detection.,

In vivo experiments: SD rats were used to establish a non‐self‐healing mandibular bone defect model. A local inflammatory environment was induced with LPS, and Bio‐Oss bone powder was applied for intervention. Two weeks after treatment, Micro‐CT and H‐E staining showed that the W54011‐loaded Bio‐Oss group exhibited better bone defect healing and denser new bone arrangement compared to the control groups. Immunohistochemical detection suggested a trend of reduced IL‐6 expression and increased osteogenic marker levels in the W54011‐treated group. However, due to the small sample size (n = 3), these differences were not statistically significant and should be interpreted as preliminary qualitative observations rather than definitive proof of superior efficacy. It should be noted that small animal bone metabolism differs significantly from that of humans, and these results should not be directly extrapolated to humans (何帆 et al. 2021).

### 4.2 Comparison with Previous Studies

Role of the C5a‐C5aR signaling axis: The C5a‐C5aR signaling axis plays a crucial role in post‐surgical bone formation (Mödinger et al. 2018). Complement C5a, an abundant innate immune factor, mediates immune cell chemotaxis (López‐Lera et al. 2019) and activates downstream inflammatory factor expression pathways via C5a‐C5aR, promoting excessive inflammatory factor release (Bosmann et al. 2013); massive inflammatory factors further activate immune cell and osteoclast differentiation, disrupting the osteogenic micro environment (Yokota 2017). Previous studies have shown two strategies to block the C5a‐C5aR axis: down regulating C5a levels or antagonizing C5aR. However, our prior research indicated that C5a benefits DPSCs differentiation, so we chose the competitive antagonist W54011 to block the axis (Wang et al. 2021b; Liu et al. 2021), which differs from studies focusing solely on C5a down regulation.

DPSCs isolation and identification: DPSCs lack unique surface molecule profiles, are scarce in dental pulp, and reside quiescently around blood vessels, proliferating, migrating, and differentiating upon activation. The academic community generally identifies DPSCs by positive CD73, CD90, CD105 expression and negative CD34, CD45 expression. Previous studies have used immunomagnetic beads for DPSCs isolation, but this requires prior cell activation and proliferation. Our group's prior research showed that culturing primary dental pulp cells with Cyagen mesenchymal stem cell medium yields high CD73, CD90, and CD105 expression via flow cytometry (Liu et al. 2021), offering a more convenient and stable method for obtaining high‐purity DPSCs. In this study, we further standardized the DPSCs culture method and only used third‐passage cells with stable morphology and proliferation for experiments, ensuring the consistency and repeatability of experimental results.

Animal models of bone defects: Most previous bone defect models were established in long bones or skulls, but the mandible has a unique developmental origin—while most skeletal tissues derive from the mesoderm (Couly et al. 1993), maxillofacial bones originate from neural crest tissue (何帆 et al. 2021). Differences in mechanical load, blood supply, oral mucosal coverage, and microbial environment also distinguish maxillofacial bone regeneration. Thus, cranial and long bone defect models are unsuitable for evaluating maxillofacial bone regeneration (Liu et al. 2019). Previous studies (e.g., Lye's polymethylacrylate biocompatibility research) used mandibular incision approaches to expose rabbit bilateral mandibular bodies (Lye et al. 2013), but this disrupts muscle integrity and model functionality. Our study established a 6 mm × 2 mm × 0.5 mm non‐self‐healing mandibular defect model in SD rats' anterior tooth region, balancing defect size and minimal impact on experimental animals' physiological structure and function.

Intervention strategies and materials: Previous studies confirmed that 4 μg/μl LPS induces rat skull inflammation (刘俊 et al. 2019); since the mandible and skull are both cortical bone, we adopted this concentration for mandibular inflammation modeling. Clinically, cranial‐maxillofacial plastic surgery often requires titanium plate/screw internal fixation, necessitating secondary removal surgery that increases patient burden (Kozakiewicz and Gabryelczak 2022). Bio‐Oss bone powder, a clinically used absorbable material, promotes bone formation and reduces bone resorption (Aludden et al. 2017), acts as a biological scaffold for platelets and osteoblasts, and minimizes donor‐site complications (Aludden et al. 2017). Micro‐CT, the gold standard for assessing bone mineral density and microstructure (Kim et al. 2021; Choi et al. 2022; Parsa et al. 2015; Oláh et al. 2022; Van Dessel et al. 2017; Theye et al. 2018), confirmed that W54011 combined with Bio‐Oss achieves bone defect repair compared to Bio‐Oss alone, expanding Bio‐Oss's application in inflammatory bone defects and providing a novel adjuvant therapy strategy for clinical inflammatory bone defect repair.

### 4.3 Mechanism Discussion

The underlying mechanisms by which W54011 promotes osteogenesis and bone defect repair in inflammatory environments may involve the following aspects:Inflammatory microenvironment regulation: As a competitive C5aR antagonist, W54011 effectively blocks the C5a‐C5aR signaling axis (Wang et al. 2021b; Liu et al. 2021), inhibiting downstream inflammation‐related pathway activation and reducing inflammatory factor expression. This suppresses immune cell and osteoclast activation, eliminating inflammation‐induced impairment of the osteogenic microenvironment and creating favorable conditions for bone formation.

Potential direct regulation of osteogenic differentiation: The W54011‐only group exhibited comparable or higher expression of osteogenic genes (Osterix, OCN, RUNX2) than the blank group, suggesting that W54011 may directly upregulate the expression of core osteogenic transcription factors (e.g., RUNX2) in addition to its anti‐inflammatory effect, thereby promoting osteogenic differentiation of DPSCs. However, the specific signaling pathway (e.g., Wnt/β‐catenin or BMP/Smad) needs to be verified by further experiments with pathway inhibitors and gene knockdown techniques. DPSCs, derived from dental pulp, maintain hard tissue differentiation ability without special stimulation (Qi et al. 2020), which may synergize with W54011's potential osteogenic effect. However, further mechanistic studies are needed to verify this observation.

Synergistic effect with Bio‐Oss bone powder: Bio‐Oss provides a biological scaffold for cell attachment (Aludden et al. 2017), while W54011 optimizes the local inflammatory microenvironment. This “scaffold support + anti‐inflammatory/osteogenic regulation” synergy enhances bone defect repair efficiency, as evidenced by improved bone volume, density, and new bone arrangement in the combined treatment group. This synergy is the key reason for the superior therapeutic effect of the combined treatment, and it also provides a new idea for the combination of biological materials and small molecule drugs in bone tissue engineering.

### 4.4 Study Limitations

Although this study initially explored the role of the W54011 in the repair of inflammation‐related bone defects, it still has the following limitations that need to be further improved in subsequent studies: Limitation in representativeness of in vitro experimental samples. In the in vitro experiments, the source of DPSCs and the number of cell preparations were limited, which failed to fully cover the heterogeneity among different donors. Due to individual differences in primary cells, limited donor samples may lead to insufficient stability and representativeness of experimental results, making it difficult to fully reflect the regulatory effect of W54011 on osteogenic differentiation of DPSCs from different sources. This limitation may affect the generalizability of the in vitro experimental results, and subsequent studies need to expand the number of DPSCs donors to at least 6 to reduce the impact of individual heterogeneity.

Limited statistical power of animal experiments: A total of 12 SD rats were included in the in vivo experiment, with only 3 rats per group (n = 3). In subsequent studies, the sample size should be increased to at least 6 rats per group to improve statistical reliability and reduce random errors. Sample size is a key factor determining statistical power; a small sample size may increase random errors, making it difficult to exclude the interference of accidental factors on the results and reducing the statistical reliability and persuasiveness of the study conclusions. In addition, this study only set a single concentration of W54011 (1 μg/ml), and subsequent studies need to set multiple dose groups to determine the optimal effective concentration and dose‐effect relationship.

Gap between animal model and clinical translation. A rat mandibular defect model was used in the experiment, and there are significant differences in bone tissue structure, healing rate, inflammatory response mechanism, and physiological environment between rats and humans. The bone healing process of small animals is relatively fast, and they lack the complex oral microecology and occlusal mechanical environment of humans, resulting in difficulty in directly extrapolating the research results to the clinical treatment of inflammation‐related bone defects.

Short study cycle. This study mainly observed the healing of bone defects in the short term (within 4 weeks) after surgery, and failed to evaluate the long‐term efficacy and safety of W54011. Bone tissue repair is a long‐term process; short‐term results cannot reflect the continuous regulatory effect of the drug on bone regeneration, nor can it monitor possible long‐term adverse reactions (such as local tissue irritation, immune rejection, etc.).

The above limitations suggest that the conclusions of this study need to be further verified in larger sample sizes, multi‐dose intervention schemes, and large animal models closer to clinical settings to provide more sufficient experimental evidence for the clinical translational application of W54011.

### 4.5 Study Significance and Future Directions

Scientific value: This study confirms that W54011 promotes DPSCs osteogenic differentiation and inflammatory environment‐induced bone defect healing, providing important scientific evidence for W54011's osteogenic role in inflammatory conditions and the first experimental evidence for the application of C5aR antagonists in inflammatory maxillofacial bone defect repair. It also clarifies the feasibility of blocking the C5a‐C5aR axis via C5aR antagonism (rather than C5a downregulation) to preserve C5a's beneficial effects on DPSCs, enriching the understanding of C5a‐C5aR axis regulation in osteogenesis and providing a new research direction for the study of complement system in bone tissue engineering.

Clinical significance: This study proposes a novel clinical strategy for bone defect treatment‐promoting osteogenesis while combating inflammation—addressing the key issue of poor post‐plastic surgery bone formation due to inflammation. The combination of W54011 and Bio‐Oss simplifies surgical procedures, reduces secondary surgery risks, and improves osteogenesis success rates, holding significant clinical application prospects for the treatment of inflammatory bone defects caused by trauma, infection, and post‐surgery in the oral and maxillofacial region.

Optimize experimental design: Expand DPSCs donor sources to reduce heterogeneity impacts; increase animal sample sizes and set multi‐dose W54011 intervention groups to determine the optimal effective concentration and dose‐effect relationship; adopt large animal models (closer to clinical settings) to enhance translational relevance; extend the experimental observation period to evaluate the long‐term efficacy and safety of W54011.

Deepen mechanistic research: Investigate the direct osteogenic mechanism of W54011 (e.g., interaction with osteogenic signaling pathways or transcription factors like RUNX2) using pathway inhibitors, gene knockdown, and immunofluorescence co‐localization techniques; explore crosstalk between the C5a‐C5aR axis and DPSCs osteogenic differentiation pathways; clarify the molecular target of W54011 in DPSCs.

Promote clinical translation: Conduct long‐term preclinical studies to evaluate W54011's efficacy and safety; verify its application in different maxillofacial bone defect types (e.g., post‐tumor resection, traumatic defects, periodontal bone defects); develop W54011‐loaded biological scaffolds with sustained release effect to improve the clinical application convenience of the drug; provide sufficient experimental evidence for clinical application.

## Author Contributions

X.T. designed the study, performed the experiments and wrote the manuscript. JH performed the experiments and organized the images. Z.C. participated in this experiment, made substantial contributions to the acquisition of data and were involved in drafting the manuscript. J.H.L. analyzed and interpreted the data and organized the images, interpreted the data. X.S. performed the surgery to obtain the teeth and was involved in revising the manuscript. W.H. and M.L. conceived and designed the study, provided funding support, revised the manuscript critically for important intellectual content, and were responsible for the final approval of the version to be published. All authors read and approved the final manuscript.

## Ethics Statement

The article proposal was approved by The Review Board of Plastic Surgery Hospital, Chinese Academy of Medical Sciences, with an ID of 2022(171). Written informed consent was obtained from all participants. The animal experiments in this study were approved by the Animal Ethics Committee of the Second Affiliated Hospital of Harbin Medical University, with the approval number ky2020‐057. All experimental procedures were performed in strict accordance with the Guidelines for Ethical Review of Laboratory Animal Welfare (Torgbo, 35892‐2018) and the 3 R principle (Replacement, Reduction, Refinement) of laboratory animal care.

## Conflicts of Interest

The authors declare that they have no competing interests. This study was not funded by any commercial organization, and there is no conflict of interest between the authors and the research content.

## Data Availability

The datasets used for the current study are available from the corresponding author on reasonable request. All raw data and experimental images of this study have been stored in the laboratory database and can be provided to relevant researchers for verification after obtaining the corresponding author's approval.
